# Two Decades of Declining Stroke Burden in Kaunas, Lithuania (2000–2023): A Population-Based Analysis of Morbidity, Mortality, and Case-Fatality Trends by Sex, Age, and Stroke Type

**DOI:** 10.3390/medicina62050824

**Published:** 2026-04-26

**Authors:** Erika Jasukaitienė, Šarūnas Augustis, Ričardas Radišauskas, Lolita Šileikienė, Abdonas Tamošiūnas, Dalia Lukšienė, Gintarė Šakalytė, Diana Žaliaduonytė, Karolina Marcinkevičienė, Daina Krančiukaitė-Butylkinienė

**Affiliations:** 1 Institute of Cardiology, Medical Academy, Lithuanian University of Health Sciences, LT-50162 Kaunas, Lithuania; sarunas.augustis@lsmu.lt (Š.A.); ricardas.radisauskas@lsmu.lt (R.R.); lolita.sileikiene@lsmu.lt (L.Š.); abdonas.tamosiunas@lsmu.lt (A.T.); dalia.luksiene@lsmu.lt (D.L.); gintare.sakalyte@lsmu.lt (G.Š.); karolina.marcinkeviciene@lsmu.lt (K.M.); daina.butylkiniene@lsmu.lt (D.K.-B.); 2 Department of Internal Medicine, Medical Academy, Lithuanian University of Health Sciences, LT-47144 Kaunas, Lithuania; 3 Department of Environmental and Occupational Medicine, Medical Academy, Lithuanian University of Health Sciences, LT-47181 Kaunas, Lithuania; 4 Department of Preventive Medicine, Faculty of Public Health, Medical Academy, Lithuanian University of Health Sciences, LT-47181 Kaunas, Lithuania; 5 Department of Cardiology, Medical Academy, Lithuanian University of Health Sciences, LT-50161 Kaunas, Lithuania; diana.zaliaduonyte@lsmu.lt; 6 Department of Family Medicine, Medical Academy, Lithuanian University of Health Sciences, LT-50161 Kaunas, Lithuania

**Keywords:** stroke incidence, stroke mortality, case-fatality, population-based registry, sex, age, Joinpoint regression, Lithuania

## Abstract

*Background and Objectives*: Stroke remains a major contributor to global morbidity and mortality, with substantial geographic variation in incidence and outcomes. Although declining trends in stroke incidence and mortality have been documented in several Western European populations, countries in Eastern Europe have historically experienced a disproportionately high cardiovascular disease burden. Comprehensive long-term evaluations assessing simultaneous trends in stroke attack rates, mortality, and case-fatality in Lithuania are limited. This study aimed to investigate 24-year trends (2000–2023) in stroke epidemiology among working-age residents of Kaunas city. *Materials and Methods*: Data were derived from the Kaunas population-based stroke registry and included individuals aged 25–64 years. Age-standardized attack rates, mortality rates, and case-fatality rates per 100,000 population were calculated using the World Health Organization standard population. Temporal trends were assessed using Joinpoint regression analysis to estimate annual percentage changes (APCs) with corresponding 95% confidence intervals (CIs). Analyses were stratified by sex, age group (25–54 and 55–64 years), and stroke subtype (ischemic and hemorrhagic). *Results:* During 2000–2023, overall stroke attack rates declined significantly in both sexes, with a more pronounced reduction observed among females. Stroke mortality decreased significantly among females over the entire study period, whereas no significant overall change was observed among males, largely due to increases during 2010–2021 that attenuated earlier and subsequent improvements. Case-fatality rates demonstrated no significant overall long-term trend in either sex but exhibited marked temporal variability, including significant increases during 2010–2021 followed by substantial declines after 2021. Age-stratified analyses confirmed significant reductions in attack rates across both age groups. Ischemic stroke incidence declined significantly in both sexes, while hemorrhagic stroke mortality decreased significantly among males and females. The period 2021–2023 was characterized by pronounced reductions in mortality and case-fatality across multiple subgroups. *Conclusions*: Over the past two decades, the stroke burden among working-age residents of Kaunas has declined substantially, particularly among females. Despite period-specific deteriorations, recent improvements underscore the impact of advances in stroke prevention and acute care. Sustained risk factor control and continued healthcare system development remain essential to maintain favourable trends.

## 1. Introduction

Stroke remains one of the leading causes of morbidity, disability, and mortality worldwide and continues to pose a substantial public health challenge, placing a considerable burden on healthcare systems [1]. Ischemic stroke is the most prevalent type, accounting for the majority of cases, while hemorrhagic stroke—despite lower incidence—is associated with substantially higher short-term case-fatality [2]. Despite well-established pathophysiological mechanisms and major risk factors, important differences in stroke incidence, mortality, and survival persist across populations, age groups, and between men and women [3,4,5].

Over recent decades, many European countries have reported declining trends in stroke incidence and mortality, largely attributed to improvements in cardiovascular risk factor control, advances in diagnostics, and the development of organized acute stroke care systems [6]. In high-income countries such as the United Kingdom, Sweden, Canada, and the United States, reductions in stroke mortality have been driven by both decreasing incidence and improved survival after stroke [7,8]. However, in parts of Eastern Europe, the stroke burden has remained comparatively high, and trends have not been uniformly favourable [6]. In Lithuania, previous analyses based on the World Health Organization MONICA project demonstrated a significant decline in stroke mortality in the working-age population of Kaunas between 1986 and 2002, while incidence trends were less consistent and differed by sex [9,10,11]. Nevertheless, comprehensive long-term evaluations covering the past two decades and simultaneously assessing incidence, mortality, and case-fatality across different stroke types remain limited. Such analyses are essential for disentangling whether changes in mortality are driven primarily by reduced occurrence, improved survival, or both.

The period from 2000 to 2023 encompasses substantial developments in stroke prevention strategies, diagnostic technologies, and acute management, including the implementation of evidence-based therapies and organized stroke care. Evaluating long-term population-based trends during this period provides critical insight into the evolving stroke burden and potential disparities across demographic groups.

Therefore, the aim of the present study was to examine trends in stroke incidence, mortality, and case-fatality in Kaunas, Lithuania, between 2000 and 2023, using population-based data, with analyses stratified by sex, age, and stroke type.

## 2. Methods

The study included all patients aged 25–64 years who experienced a stroke between 2000 and 2023 and were permanent residents of Kaunas city. Stroke registration methods followed the protocol laid down in the WHO (World Health Organization) MONICA (Multinational MONItoring of trends and determinants in Cardiovascular disease) project [12].

The retrospective data collection was used to identify stroke events. Multiple sources of information were used for case ascertainment, including hospital discharge records, medical files, outpatient records, medical death certificates, and autopsy reports [13,14].

According to the WHO MONICA protocol [14,15,16], stroke was defined as “rapidly developed clinical signs of focal (or global) disturbance of cerebral function lasting more than 24 h (unless interrupted by surgery or death) with no apparent cause other than a vascular origin”.

Global symptoms apply to patients with coma or subarachnoid hemorrhage without focal neurological signs. Multiple stroke attacks occurring within 28 days of the onset of the symptoms of the first attack were considered as one event.

A stroke event was defined as fatal if death occurred within the first 28 days from onset. If the patient was alive after 28 days, the stroke was classified as non-fatal. The specific type of stroke was confirmed by dedicated diagnostic examinations. For subarachnoid hemorrhage (SAH), brain CT, sampling of cerebrospinal fluid containing blood, or autopsy were required to establish the diagnosis; for intracerebral hemorrhage (ICH), confirmation was required by the brain CT or autopsy. Ischemic stroke was diagnosed when CT and/or autopsy could verify brain infarction and/or exclude hemorrhage and non-vascular disease.

The study was approved by the Lithuanian Bioethics Committee (ref. No. 14-27/03 December 2001) and the Kaunas Regional Biomedical Research Ethics Committee (ref. No. BE-2-39/19 April 2021). All patient records/information were anonymized and de-identified before the analysis.

### Statistical Analysis

Stroke attack and mortality rates were calculated per 100,000 population using the world standard [12]. Changes in attack and mortality rates were assessed using linear regression through the Joinpoint regression analysis tool [13], and the results are presented as annual percentage changes (APCs) with corresponding 95% confidence intervals (CIs). Joinpoint regression analyses for the 25–64 years age group were performed to assess changes in the annual sex-specific attack and mortality rates over the study period. Joinpoint regression was conducted using segmented log-linear models fitted via weighted least squares estimation, whereby weights are derived from the variance of age-standardized rates. This approach accounts for heteroscedasticity inherent in rate data and provides stable estimation across years with varying population denominators. Joinpoint analysis is a data-driven statistical method that detects inflexion points in trends and fits multiple linear regression lines, based on a predefined number of joinpoints (junction points) [17]. Since there were 24 points in total, a maximum of three junction points were selected for analysis. Initially, a zero-junction-point model was chosen. The final number of joinpoints (0–3) was determined using the Monte Carlo permutation test, which compares model fit across competing segmented models and selects the most parsimonious model that achieves statistically significant improvement. Model adequacy was evaluated using weighted error statistics (sum of squared errors and mean squared error) and visual inspection of observed versus fitted values, as recommended by the NCI Joinpoint technical documentation. The absence of autocorrelation in residuals, as indicated by the Joinpoint autocorrelation test, further supported appropriate model fit. No patterns suggestive of problematic overdispersion were observed. A two-sided *p*-value < 0.05 was considered statistically significant.

## 3. Results

**Figure 1:**
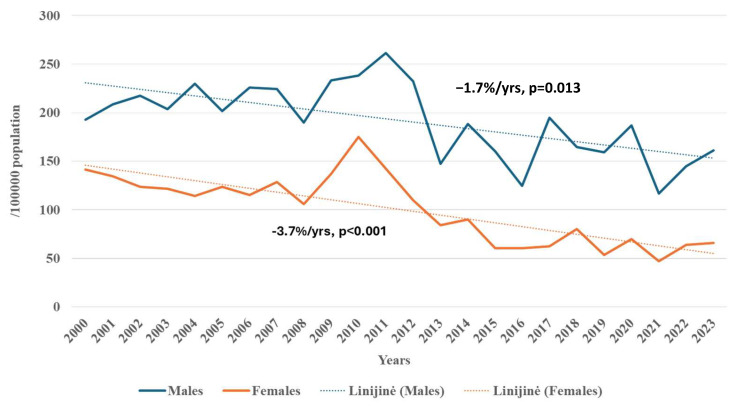
Trends in the attack rate of stroke among males and females aged 25–64 in Kaunas in 2000–2023.

**Figure 2:**
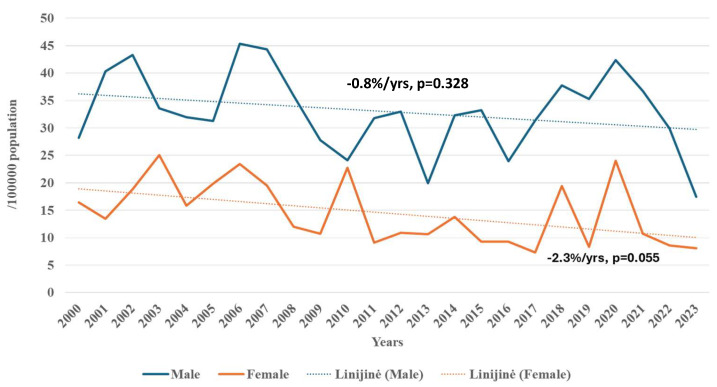
Trends of mortality from stroke among males and females aged 25–64 in Kaunas in 2000–2023.

From 2000 to 2023, the attack rates of stroke among Kaunas city residents aged 25–64 years significantly decreased both in females and males (Figure 1 and Figure 2). A significant decline in stroke attack rates was observed in females during 2010–2015 and in males during 2010–2023. However, among females, a significant increase in stroke attack rates occurred between 2007 and 2010.

Mortality rate from all strokes significantly decreased in females over the entire 24-year study period, while no significant change was observed in males overall. Among females, notable declines in mortality rate from all strokes were also recorded during 2003–2016 and 2020–2023. In male residents of Kaunas city, significant reductions in mortality rate from all strokes were observed during 2007–2010 and 2021–2023. However, from 2010 to 2021, the average increase in APC for mortality from all strokes in males was +3.9% (*p* < 0.05).

**Figure 3:**
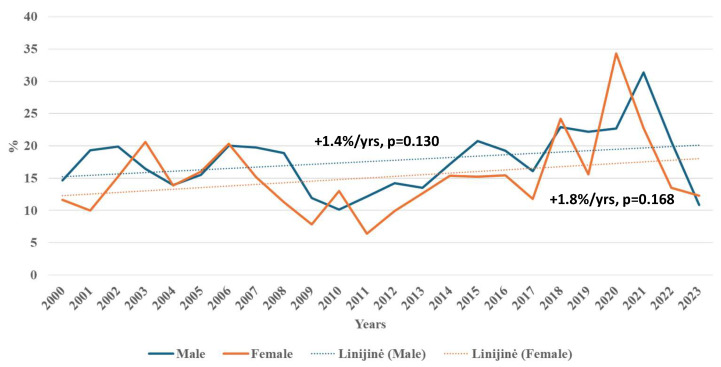
Trends of fatality from stroke among males and females aged 25–64 in Kaunas in 2000–2023.

The case-fatality rate of all strokes showed no significant change in either sex when the entire 24-year period was analyzed. However, among female Kaunas city residents, significant decreases in case-fatality from all strokes were observed during 2006–2011 and 2020–2023. In males, a significant decrease was also noted from 2021 to 2023. Conversely, increasing trends in case-fatality from all strokes were found during 2011–2020 in females and 2010–2021 in males (Figure 3).

Age-stratified analyses demonstrated declining stroke attack rates in both the 25–54 and 55–64 year age groups for males and females, though some important age- and sex-specific variations were observed.

Among 25–54-year-old males, stroke attack rate declined significantly over the study period (APC −1.59%, *p* < 0.05), with the most pronounced decline occurring after 2011 (APC −4.95%, *p* < 0.05). Mortality and case-fatality rates exhibited substantial temporal variability, including significant increases during 2000–2007 and 2010–2021, followed by dramatic declines during 2021–2023. Mortality rate decreased by 55.7% (*p* < 0.05) and case-fatality rate by 48.4% (*p* < 0.05) during this recent period. Among males aged 55–64 years, attack rates declined significantly overall (APC −1.70%, *p* < 0.05), particularly after 2007 (APC −3.10%, *p* < 0.05). In both male age groups, mortality and case-fatality rates showed no significant overall changes during the study period.

For females aged 25–54 years, attack rates decreased significantly (APC −3.29%, *p* < 0.05), with accelerated decline after 2010 (APC −9.33%, *p* < 0.05). Mortality and case-fatality demonstrated marked temporal fluctuations, with significant increases during 2000–2003 followed by declines during 2003–2016. Notable reductions in both mortality (APC −41.1%, *p* < 0.05) and case-fatality rates (APC −36.6%, *p* < 0.05) were observed during 2020–2023. Among females aged 55–64 years, stroke attack rates declined significantly overall (APC −3.94%, *p* < 0.05), particularly during 2010–2021 (APC −8.45%, *p* < 0.05). However, a noteworthy significant increase in attack rates was observed during 2021–2023 (APC +28.8%, *p* < 0.05). Mortality decreased significantly over the entire period (APC −2.51%, *p* < 0.05), while case-fatality showed no significant overall change but exhibited some period-specific variations.

Furthermore, trends differed by stroke type, with both ischemic and haemorrhagic strokes showing overall favourable patterns despite several period-specific variations.

Ischemic stroke attack rate among individuals aged 25–64 years declined significantly over the study period in both males (APC −1.74%, *p* < 0.05) and females (APC −4.30%, *p* < 0.05). Among males, a non-significant increase was observed during 2000–2010, followed by significant declines thereafter (APC −4.43%, *p* < 0.05). Among females, rates decreased significantly during 2000–2007, then increased sharply during 2007–2010 (APC +22.7%, 95% CI: 8.12 to 31.3, *p* < 0.05), followed by a substantial decline during 2010–2015 (APC −21.4%, *p* < 0.05), after which rates stabilized. Mortality from ischemic stroke among individuals aged 25–64 years showed no significant overall change in either sex. Case-fatality increased significantly over the entire period in both males (APC +2.45%, *p* < 0.05) and females (APC +3.64%, *p* < 0.05), but showed dramatic recent declines during 2020–2023 in females (APC −33.7%, *p* < 0.05) and 2021–2023 in males (APC −34.9%, *p* < 0.05).

Haemorrhagic stroke (including intracerebral and subarachnoid hemorrhage) attack rate among individuals aged 25–64 years declined significantly in males (APC −1.69%, *p* < 0.05) but showed no significant overall change in females (APC −1.60%, *p* = 0.1). Mortality from haemorrhagic stroke decreased significantly in both males (APC −3.08%, *p* < 0.05) and females (APC −3.49%, *p* < 0.05). Case-fatality showed no significant overall change in either sex but demonstrated period-specific variations, including significant declines during 2021–2023 in both males (APC −27.0%, *p* < 0.05) and females (APC −43.3%, *p* < 0.05).

## 4. Discussion

Overall, stroke attack rates declined significantly in both sexes, with females showing a more pronounced reduction compared to males. Mortality from all strokes decreased significantly in females but showed no significant overall change in males, reflecting a significant increase during 2010 and 2021 that offset earlier and later improvements. Case-fatality exhibited no significant overall trend in either sex but demonstrated marked period-specific fluctuations, including increases during 2010–2021 followed by pronounced declines after 2021. The pronounced reductions in mortality and case-fatality observed in 2021–2023, however, should be interpreted with caution, as small annual numbers and pandemic-related fluctuations may contribute to short-term instability in rate estimates.

These findings extend previous observations from the Kaunas stroke registry covering the earlier period of 1986–2012 [11]. During that earlier period, stroke mortality rates declined significantly in both sexes, while stroke incidence showed no trends in males and an increasing trend in females. The current analysis (2000–2023) demonstrates a sustained decline in mortality trends, particularly pronounced in females.

Our findings align with broader European trends showing declining stroke incidence and mortality over recent decades, though the magnitude and timing of changes vary considerably across populations [18]. Several well-established European stroke registries provide valuable context for interpreting the Kaunas data.

The Dijon Stroke Registry in France reported declining stroke incidence among individuals aged 45–64 years (rather than 25–64, given the rarity of stroke in younger adults in Western European populations) between 1987 and 2011, with particularly pronounced decreases in haemorrhagic stroke [19,20]. Similarly, the Oxford Vascular Study in the United Kingdom demonstrated significant reductions in stroke incidence between 2002 and 2013, attributed to improved primary prevention and risk factor control [21]. The Erlangen Stroke Project in Germany documented declining stroke incidence during the 1990s and early 2000s, primarily driven by reductions in haemorrhagic stroke, consistent with improved hypertension management [22].

Data from Northern European countries suggest similar patterns. Swedish national registry data indicated declining stroke incidence and mortality over the past two decades, with particularly favourable trends in ischemic stroke reflecting widespread use of evidence-based preventive therapies [23,24]. Finnish studies have reported comparable reductions, especially among younger age groups, attributed to comprehensive population-level cardiovascular risk factor control programmes [25].

However, the Kaunas data differ from Western European registries in several important perspectives. Baseline stroke rates in Kaunas at the beginning of the study (year 2000) were substantially higher than those reported in Western Europe, reflecting Lithuania’s historical classification among high cardiovascular disease burden countries [11,26]. The temporal pattern in Kaunas Stroke Registry –characterized by initial improvements followed by deterioration during 2010–2021 and then more recent gains—differs from the more consistently declining trends observed in many Western European populations [18].

Eastern European data remain relatively sparse, but available studies from Poland, the Czech Republic, and Estonia suggest patterns more similar to Kaunas, with high baseline rates, variable trends during the 2000s and 2010s, and more substantial recent improvements as stroke care infrastructure has modernized [27,28,29]. This regional pattern likely reflects shared historical context, progressive healthcare system development, and gradual but continuous implementation of evidence-based stroke care, including the establishment of stroke care networks and regional stroke centres. Giving the context of the most recent global estimates to our data provides an important perspective. The GBD 2021 study reported that globally, age-standardized stroke incidence and mortality have declined since 1990, yet noted a stagnation in incidence reduction from 2015 onwards, with some regions experiencing increases—a pattern consistent with the non-linear temporal trends observed in the present study [30].

An important structural factor contributing to improved stroke outcomes in the present study is the comprehensive national acute stroke care policy implemented in Lithuania in 2014. This policy has been demonstrated to significantly improve a wide range of immediate and long-term stroke care quality measures, including access to organized stroke unit care, thrombolysis rates, and adherence to evidence-based management protocols. The progressive implementation of the national stroke care system likely contributed to the sustained improvements in mortality and case-fatality observed, particularly after 2021, and may partly explain the more favourable trends observed in the present study compared with the earlier 1986–2012 period.

Several potential mechanisms must also be considered in the interpretation of the temporal patterns of Kaunas data. Changes in cardiovascular risk factor prevalence during the study period must have contributed [31,32]. Lithuania experienced significant socioeconomic transitions following European Union accession in 2004, potentially affecting lifestyle, including dietary habits, physical activity, etc. [33]. Unfortunately, the temporal pattern coincides with the global financial crisis of 2008–2009 and its aftermath as well. Economic stress has been associated with increased cardiovascular risk through multiple pathways, including psychological stress, medication non-adherence, and adverse health behaviours [34,35]. However, we must acknowledge that these explanations remain largely speculative in the absence of individual-level data on risk factor prevalence, treatment adherence, healthcare utilization, and socioeconomic status across the study period. Without such data, causal attribution remains inferential rather than evidence-based, and these explanations should be interpreted as hypothesis-generating rather than conclusive. Moreover, healthcare system evolution may have influenced case detection and ascertainment. Enhanced diagnostic capabilities, including universal availability of computed tomography (approaching 100% of suspected stroke cases) and increased utilization of magnetic resonance imaging, could have improved identification of milder strokes that previously went undetected [36,37].

The distinct trends between stroke types reflect distinct pathophysiological mechanisms and risk factor profiles. Declining ischemic stroke rates, particularly in older age groups, align with improved lipid management and cumulative lifestyle modifications targeting atherosclerotic risk factors. Haemorrhagic stroke trends, characterized by more pronounced declines in mortality and case-fatality, are primarily driven by hypertension and stress management, with particular improvements observed in younger populations and among women. Recent population surveys demonstrate enhanced blood pressure control and treatment adherence, especially among Lithuanian females [38,39], which likely contributes to the more favourable haemorrhagic stroke trends in the female population.

The COVID-19 pandemic, beginning in early 2020 and extending through the final years of this study, most clearly exacerbated existing challenges. Delayed presentation due to fear of hospital-acquired infection, healthcare system strain and resource reallocation, and disruption of regular follow-up care contributed to the peak case-fatality rates observed in 2020–2021 [40]. Furthermore, the COVID-19 infection itself has been associated with increased stroke risk through multiple pathways, including hypercoagulability, endothelial dysfunction, and inflammatory responses [41,42,43]. The prothrombotic state induced by severe COVID-19 infection may have contributed to stroke occurrence, particularly during peak infection waves [44,45].

Paradoxically, while the COVID-19 pandemic increased stroke incidence, contributed to more severe disease courses, and created substantial healthcare challenges, it also catalyzed innovations that may have contributed to the improvements in stroke outcomes observed during 2021–2023. The rapid deployment of telemedicine infrastructure enabled remote stroke consultations and improved coordination between primary hospitals and comprehensive stroke centres [46]. Enhanced emergency services protocols, implemented to manage pandemic-related emergencies, may have simultaneously improved stroke triage and transport [47]. Greater health consciousness following the pandemic, including increased awareness of cardiovascular risk factors and the importance of timely medical attention, may have also facilitated earlier stroke recognition and presentation.

The more favourable long-term trends observed among females compared with males—characterized by significantly declining attack rates and mortality in females versus stable overall mortality in males—likely reflect multiple interconnected factors, including differences in risk factor profiles, healthcare utilization patterns, biological susceptibility, and age-related patterns, with more pronounced improvements observed among younger females (25–54 years) compared to their male counterparts [32].

This study has several important strengths that enhance confidence in the findings and the interpretations. The long-term observation spanning 24 years (2000–2023) represents one of the longest continuous population-based stroke surveillance periods reported, enabling detection of both long-term trends and period-specific variations [11]. The restriction to the working-age population (25–64 years) is important, as stroke in this demographic carries substantial socioeconomic implications through premature mortality, disability, and lost productivity. The observed trends therefore reflect changing stroke burden among economically active residents of Kaunas city during their productive life years. The use of a well-established population-based stroke registry with standardized case-ascertainment methodology ensures high validity and comparability of data across the study period. The application of Joinpoint regression analysis represents a methodologically rigorous approach to trend detection, enabling data-driven identification of temporal inflexion points. Furthermore, detailed stratification by sex, age group, and stroke type provides fine-level information on differential trends across population subgroups.

### Limitations

A few important limitations must also be considered. Although the restriction of analyses to individuals aged 25–64 years limits generalizability to older populations, where the majority of strokes occur, this approach was maintained to ensure methodological consistency with the long-standing stroke registry protocol [4]. The lack of individual-level data on risk factors, treatment modalities, and stroke severity somewhat limits the causal interpretation of observed trends. Changes in diagnostic practices and imaging availability over the 24-year study period may have influenced case ascertainment and classification [25,26]. The study cannot distinguish between first-ever and recurrent strokes, as overall attack rates include both incident and recurrent events. This limits interpretation of whether observed trends reflect changes in primary stroke occurrence, recurrence rates, or both, and may introduce some heterogeneity into the trend estimates. Further studies incorporating artificial intelligence-based tools for automated case classification and electronic health record linkage could enable systematic differentiation between first-ever and recurrent stroke at the population level, providing deeper insight into the independent contributions of incidence and recurrence to overall stroke burden trends.

The study cannot distinguish between first-ever and recurrent strokes. The study covers a single city and may not be representative of rural areas or other regions of Lithuania. The integration of individual-level data on vascular risk factors, treatment patterns, stroke severity, and acute management strategies would enable a more detailed assessment of determinants underlying temporal trends. Nationwide multicentre registry studies, including both urban and rural populations, are needed to evaluate regional variations in stroke epidemiology across Lithuania and would provide valuable evidence to strengthen both primary and secondary stroke prevention strategies in the country.

## 5. Conclusions

Over the past two decades, the stroke burden among working-age residents of Kaunas city has undergone substantial and complex changes. Overall stroke attack rates declined significantly in both sexes, with females experiencing greater reductions than males. Despite periods of concerning deterioration, particularly during 2010–2021, the recent improvements after 2021 demonstrate the potential of modern evidence-based stroke care to transform outcomes. The substantial reductions in mortality and case-fatality among younger adults during 2021–2023 represent the successful implementation of comprehensive stroke care.

## Data Availability

All relevant data are within the paper.
